# Efforts, rewards and professional autonomy determine residents’ experienced well-being

**DOI:** 10.1007/s10459-018-9843-0

**Published:** 2018-08-07

**Authors:** S. S. Lases, Irene A. Slootweg, E. G. J. M. Pierik, Erik Heineman, M. J. M. H. Lombarts

**Affiliations:** 1 Professional Performance Research Group, Department of Medical Psychology, Academic Medical Center, University of Amsterdam, Meibergdreef 9, PO Box 22660, 1100 DD Amsterdam, The Netherlands; 2 Department of Surgery, Isala, Zwolle, The Netherlands; 3 Department of Public Health and Primary Care, Leiden University Medical Center, Leiden, The Netherlands; 4 Department of Surgery, University Medical Center Groningen, Groningen, The Netherlands

**Keywords:** Residents’ experienced well-being, Postgraduate medical education, Professional life, Influencing factors, Effort–reward balance, Professional autonomy

## Abstract

The well-being of residents, our future medical specialists, is not only beneficial to the individual physician but also conditional for delivering high-quality patient care. Therefore, the authors further explored how residents experience their own well-being in relation to their professional and personal life. The authors conducted a qualitative study based on a phenomenological approach. From June to October 2013, 13 in-depth interviews were conducted with residents in various training programs using a semi-structured interview guide to explore participants’ experience of their well-being in relation to their professional life. The data were collected and analyzed through an iterative process using the thematic network approach. Effort–reward balance and perceived autonomy were dominant overarching experiences in influencing residents’ well-being. Experiencing sufficient autonomy was important in residents’ roles as caregivers, as learners and in their personal lives. The experienced effort–reward balance could both positively and negatively influence well-being. We found two categories of ways that influence residents’ experience of well-being; (1) professional lives: delivering patient care, participating in teamwork, learning at the workplace and dealing with the organization and (2) personal lives: dealing with personal characteristics and balancing work–life. In residents’ well-being experiences, the effort–reward balance and perceived autonomy are crucial. Additionally, ways that influence residents’ well-being are identified in both their professional and personal lives. These dominant experiences and ways that influence well-being could be key factors for interventions and residency training adaptations for enhancing residents’ well-being.

## Introduction

Residents’ well-being is crucial in health care. The well-being of residents, our future medical specialists, is not only beneficial to the individual physician but is also a condition for delivering high-quality patient care (Wallace et al. 2009; Prins et al. 2009; Scheepers et al. 2015). Residents’ own experience of their well-being and its influencing factors in their professional life are relatively unknown and are therefore useful and important to further explore.

Multiple terms and constructs are used to describe well-being. Wallace uses the term wellness and describes this as “the complex and multifaceted nature of physicians’ physical, mental and emotional health and wellbeing” (Wallace et al. 2009). He also captures the positive side of being well and includes being challenged, thriving and achieving success in various aspects of personal and professional life. Various studies have shown that residents who report higher levels of well-being make fewer medical errors, deliver less suboptimal patient care and achieve higher patient satisfaction levels (Prins et al. 2009; Kim et al. 2004; Williams et al. 2007). More specifically, residents’ energy, enthusiasm and dedication to their work, their so called work engagement, appears to positively influence their professional performance (Prins et al. 2009; Scheepers et al. 2015; Mache et al. 2013). Highly engaged residents seem to display more adequate patient safety behaviors and report fewer medical errors (Prins et al. 2009; Biddison et al. 2016). Physicians who are satisfied with their jobs and are empathic not only make fewer medical errors but also have patients that are more compliant and satisfied with their care (Williams et al. 2007; Kim et al. 2004; West et al. 2006). In a systematic review of Scheepers et al. it was confirmed that physicians’ occupational well-being, including residents’ well-being, could contribute to better patient satisfaction and interpersonal aspects of patient care (Scheepers et al. 2015).

However, in modern health care, residents face various challenges within the medical profession as well as in residency training, which can impact their well-being. Dealing with the many job demands, including consistently delivering high-quality patient care, working irregular hours, being dependent on supervisors and maintaining a good work–life balance, appears to be one of the greatest challenges for residents (Prins et al. 2007; Thomas 2004). These challenges include more than only the number of hours (or duty hours) worked, as evidenced by Taylor who showed that fatigue is still commonly reported after the introduction of the regulation of work hours for residents in European countries (Taylor 2017). Additionally, the study by Hurst et al. on first-year residents showed that low team support, few learning opportunities and poor orientations are also factors decreasing residents’ well-being (Hurst et al. 2013).

The inability to react and cope successfully with the many job demands in residents’ professional lives may lead to impaired well-being with negative personal and professional consequences (Wallace et al. 2009; Shapiro et al. 1998; Hurst et al. 2013; Taylor 2017). The consequences on a personal level are illustrated by studies reporting high levels of residents’ fatigue, stress, anxiety, depression and burnout (Wallace et al. 2009; Prins et al. 2010; Govardhan et al. 2012; Guido et al. 2012; Cohen and Patten 2005; Peterlini et al. 2002; Shanafelt et al. 2015), while the negative professional consequences are expressed in reduced levels of patient satisfaction, increased medical errors and suboptimal patient care (Prins et al. 2009; Wallace et al. 2009; Shanafelt et al. 2002; West et al. 2006; Fahrenkopf et al. 2008; Kim et al. 2004; Neumann et al. 2011; Mercer and Reynolds 2002).

Residents’ well-being and its consequences have been described mostly using quantitative approaches. A few authors published their qualitative explorations on this topic. Taylor describes residents’ experiences with fatigue and the influence of duty hour regulations, and Hurst describes well-being experiences of first-year residents using qualitative research (Taylor 2017; Hurst et al. 2013). Still, residents’ own experience of their overall well-being in relation to their professional activities and conditions during residency is relatively unknown. This study aims to reduce this gap by exploring residents’ lived experiences of well-being from residents in different training levels using qualitative research (Fossey et al. 2002).

The research question leading this study is: how do residents experience their own well-being in relation to their professional life? With this study we aim to add to the existing body of knowledge of residents’ well-being for the purpose of delivering high-quality care.

## Methodology

We conducted a phenomenological study using in-depth interviews. The purpose of the phenomenological approach is to understand the essence of the phenomenon—here, well-being—from the perspective of those who have experienced it (Starks and Trinidad 2007; Ng et al. 2013; Willig 2013; Wojnar and Swanson 2007). Descriptive phenomenology is especially suitable for exploring the phenomenon through direct interaction between the researcher and the objects of study, and one key element is viewing the person as a representative of the world in which he or she lives (Wojnar and Swanson 2007; Benoist 2003). As our aim is to deepen our insights into the lived experiences of residents regarding their well-being in their professional lives, the descriptive phenomenological approach fits our aim.

Each part of the investigation contains a mix of bracketing, analyzing, intuiting and describing to create an understanding of the phenomenon under study (Swanson-Kaufmann and Schonwald 1988; Willig 2013). We will describe these steps in the following parts of this methodology section.

### Participants

Residents were invited to voluntarily participate in the study by mail through the hospital-specific resident associations and through faculty and members of the research team. The mailing explained the purpose and procedure of the study. To capture a wide and relevant variety of experiences, residents from different backgrounds were selected by purposeful sampling taking into account the residents’ year of training, gender, teaching sites and residency training programs (Kuper et al. 2008). Thirteen residents, six women and seven men, participated in the study. The participants’ ages ranged from 26 to 34 years and their year of residency from first to fourth. They attended different residency training programs, representing three university hospitals and four non-university teaching hospitals.

### Data collection

The in-depth interviews were conducted by one researcher (SSL) from June 2013 to October 2013. A semi-structured interview guide was used to explore participants’ experiences and feelings in regard to their well-being and its relation with professional activities and conditions as well as with residents’ personal lives (see “Appendix” for interview guide).

The initial interview guide was developed based on the literature on residents’ well-being and ample discussions within our research team (Wallace et al. 2009; Prins et al. 2007). The initial guide was adapted multiple times after discussing pilot interviews one and two and checked again after the third interview. In line with our phenomenological approach we focused on the personal experiences and feelings of the participants and encouraged them to describe these in their own words. The interviews were conducted in a place of the resident’s choice (home or office) to encourage the participants to speak freely. The interview time was generally one hour. The interviews were audio-recorded, and the transcripts were anonymized and transcribed verbatim. The interviews were also summarized and sent to the participants to check their correctness and allow for any additional comments, experiences or questions. Interview number 11 did not result in any new information on residents’ well-being experiences in relation to their professional life. After considering the items as suggested by Malterud et al. to determine the information power of an interview sample, namely the (1) study aim, (2) established theory, (3) sample specificity, (4) quality of dialogue and (5) analysis strategy, we felt indeed confident to stop after 13 interviews (Malterud et al. 2015; Varpio et al. 2017). We took in consideration the applied theory in the study and the study aim that is neither especially broad nor narrow. Also, the analysis strategy meant to explore experiences and patterns relevant for the study aim and the specific type of participants were weighed. Finally, we considered the fact that the researcher is a relatively novice researcher, however with thorough experience with the matters in question and (after interview training) able to communicate clearly and establish a good dialogue. These items taken into account confirmed our confidence to stop interviewing after 13 interviews (Malterud et al. 2015).

### Reflexivity

The researcher inevitably influences the research process, which makes reflexivity an essential element (Finlay 2002; Guillemin and Gillam 2004; Mantzoukas 2004). In this study, the first author and lead researcher is a surgical resident herself, making her an “insider” in regard to the topic under study and the target group. In many ways this was an advantage as this gave her an excellent understanding of the study subjects and their work contexts. We think this has facilitated the interviewees’ willingness to open up and share their professional and personal experiences (Dwyer 2009; Asselin 2003). Clearly, being an insider also comes with some challenges, in particular bringing one’s own unnoticed preconceptions to interviewing and (thus) influencing the data collection and ultimately the findings of the study (Fischer 2009; Wall et al. 2004). Being aware of researcher engagement and in striving to be as open as possible to the participants’ voices, the lead researcher tried to put aside her prior understanding or preconceptions about the phenomenon under investigation. For this process of bracketing we took some of the precautions as described in the literature (Wall et al. 2004; Fischer 2009). The researcher used a “diary” to make notes about her interview observations, her assumptions, research group meetings and any feelings of confusion. Additionally, the researcher sought advice from the research group and experts on the obtained insights to clarify her view by jointly re-listening to (parts of) the first two audio-recorded interviews and discussing the role of the interviewer and her interpretations of the residents’ responses. The other researchers had more distance from the research setting, which supported her in seeing the data from a non-insider perspective. We discovered favorable ways of phrasing questions and personal response interpretations, and this awareness indeed improved the researcher’s interview technique (Finlay 2002; Asselin 2003). This additionally enabled her to dig deeper and ask beyond what seemed to be the shared understanding. Herein, the intuitive process played an important role. At all times, the researcher tried to stay curious in regard to what it might be like to “be in the shoes of the participant”. She was aware of the importance of attentive listening in the interviews and of deep critical reflection on commonalities between participants.

Given the pervasive stigma around mental and physical health issues, the resident–residents interview structure and the familiarity between the interviewer and some of the participants might have served as a barrier to the open and honest sharing of experiences. To preempt this as much as possible, we emphasized at the beginning of the interview the anonymity of all information shared by the participant and also explained that participants would be given the opportunity to check the correctness of the interview’s summary afterwards.

### Coding and analysis

The researcher has read and re-read the interviews to get a global understanding of the participants’ experiences. After that, all interviews were coded by one researcher (SSL) using the qualitative data analysis software MAXQDA Version 11. Noticeable information on residents’ well-being was extracted and categorized into ways that influence residents’ well-being. To discuss and deepen the meaning of the data, another researcher (IAS) also coded interview numbers three and ten. Differences in coding were discussed until agreement was reached; agreement increased over time. To analyze the interviews we used thematic network analysis. This analysis tries to bring forth the experiences at different levels and aims to facilitate the organization and rich description of the data (Attride-Stirling 2001). The interviews, the way of coding and the template were discussed regularly within the research team to increase completeness and to fit the contents with the coding.

Throughout the iterative process of data collection, data coding and data analysis, the discussions in the research team continued. This contributed to gaining a deeper understanding of the data and uncovering our final insights into residents’ well-being.

### Ethical considerations

The participation was voluntary, and all participants were considered to have full control over whether they wanted to participate and to disclose personal information. To protect the anonymity and confidentiality of the study participants we took several precautions. Verbal and audio-recorded consent to use the data for research purposes was obtained before starting the interviews. Additionally, the interview transcripts were anonymized and transcribed verbatim. The institutional ethical review board of the Academic Medical Center of the University of Amsterdam waived ethical approval.

## Results

During data analysis we identified two striking overarching experiences that pervade the ways that influence well-being: the effort–reward balance and perceived autonomy. We will first explain these overarching experiences. After that, we will continue with describing the ways that influence residents’ well-being in both their professional and personal lives.

### Overarching experiences

#### The effort–reward balance

I’ve had a busy period with double tasks and long working hours. This requires a lot of effort from me. Especially when you don’t receive feedback about what you’re doing well. […..] A compliment could get me motivated and give me energy. This makes me able to perform for the upcoming weeks.

The balance between efforts and rewards was noted by residents to be important in experiencing well-being. The rewards mentioned included, for example, receiving adequate supervision and appreciation from faculty, getting support from peers, learning new things, the feeling of meaning something to a patient and having satisfied patients. The residents described these rewards as energy sources. The residents experienced having to exert extra effort in regard to aspects such as working irregular hours, conflicts with supervisors, dealing with the emotional and mental impact of suffering patients, having to make difficult decisions and having to spend free time on preparing residency-related assignments. The balance between these stressful experiences and rewards seems to impact residents’ well-being. For example, residents who experience inadequate supervisory support and incomprehension from peers report experiencing higher levels of stress. One resident even admitted these aspects in combination with a different expectation of her job content as the major reason for quitting residency training. On the other hand, when residents experience a heavy workload because of a difficult and busy consultation day at the outpatient clinic, the rewards of appreciation or compliments from a supervisor and satisfied patients could partly relieve the (temporary) distress experienced. Additionally, residents expressed the ability to handle busy training periods because they feel energized by their work and they feel that they can be of value and are appreciated.

#### Perceived autonomy

I especially like working at the outpatient clinic because I can see and treat patients myself. This in combination with an easily accessible supervisor to consult when something is not clear to me.

The feeling to be able to make (relatively) autonomous decisions was recurrently mentioned by residents as a factor important for experiencing well-being. Its importance became clear in the different resident roles as a caregiver, as a learner and also in private life. Residents noticed that making decisions about a patients’ treatment is often experienced as meaningful and educational. Getting the possibility to do so in a comfortable and entrusted way positively influenced their well-being because the residents felt fulfilled and appreciated. However, making difficult decisions can be challenging. One resident noted that making decisions about life and death is radical, and he experienced this as one of the greatest mental burdens of his job. The support of supervisors herein is essential.

A residents’ ability to influence his or her residency training schedule resulted in a feeling of being engaged and having control over the decisions in one’s life, which enhanced the experienced well-being. Also, being provided with the opportunity to set their own personal learning goals, choose their own research topic or take a break for traveling gave the residents freedom to personalize their own residency training and positively impacted their well-being. When talking about work–life balance, the residents mentioned feeling good when they were able to decide by themselves what to do in their spare time. Mandatory assignments or preparing for presentations were enjoyed less when they had to be done beyond working hours.

### Ways that influence residents’ well-being

Given the expressed residents’ experiences we can now describe the various ways that influence residents’ well-being. How residents experienced these ways that influence their well-being appeared to reflect their overall experienced well-being. The duration of a positive or negative experience had an important contribution herein. From the experienced ways that influence well-being we identified different ways in both professional and personal lives: delivering patient care, participating in teamwork, learning at the workplace, dealing with the organization, dealing with personal characteristics and balancing work–life. Table 1 contains an additional overview of these ways that influence residents’ well-being with the accessory topics described by the residents, and we will further explain these in the following text.

**Table 1:** Ways that influence residents’ well-being in both professional and personal lives, including the accessory topics described by the residents

*In professional lives*	
Delivering patient care	Patient contact
	Complications and errors
	Making complex decisions
	Workload
Participating in teamwork	Supervisors
	Peers
	Multidisciplinary work
Learning at the workplace	Work and learning climate
	Competency development
	Courses and assignments
	Assessments and feedback
Dealing with the organization	Duty hours
	Logistics
	Administration
	Bureaucracy
*In personal lives*	
Dealing with personal characteristics	Personality
	Power and pitfalls
	Coping strategies
Balancing work-life	Time distribution
	Family and friends
	Sports and activities

### In professional lives

#### Delivering patient care

I receive energy from dealing with patients in the right way; explaining the situation and what is going on with an ill patient in order to make the patient and family understand what is happening, that gives me a feeling of satisfaction.

Having meaningful contact with patients or patients’ families gives residents energy and a feeling of satisfaction. The fascination with the human body and being allowed to be a part of the intense emotional live of patients is experienced as unique and beautiful. The residents mentioned being energized from seeing a variety of patients with different kinds of problems. Additionally, they expressed enjoying the social aspect of patient contact and experience patients being satisfied as real energy boosters. On the other side, the residents also describe confronting emotional situations with ill patients, which can negatively influence their experience of well-being for a while. The residents report that experiencing complications or weighing a case from different perspectives and making significant decisions about treatment options can be emotionally and mentally challenging. In these cases they find it particularly difficult to let go of work-related thoughts when leaving the hospital. Thinking about patients may continue at night and may sometimes lead residents to call the department to check on the patients’ actual situation. The workload can be experienced as heavy when residents have to see too many (complicated) patients in the outpatient clinic, when they have too many tasks at the same time or when they are continuously being disturbed by a ringing phone. This can leave them feeling overwhelmed or agitated or cause them to lose concentration and overview. However, residents like being challenged with a variety of tasks and patient cases in order to learn and feel satisfied.

#### Participating in teamwork

I feel supported by my supervisor. He is sometimes a little more precise than I am, and it is pleasant to deliberate about and discuss a patient. This results in a collective feeling that you have formed the best plan for the patient. That creates a good feeling.

The relation to and connection with supervisors is an important influencing factor for residents’ well-being. When the residents experience receptiveness and support in decision-making, they feel more confident and appreciated. Alternatively, conflicts with supervisors or supervisors not being available for deliberation, negatively influences the residents’ well-being: it makes them feel anxious and insecure. Additionally, it diminishes their work satisfaction. Working together with peers has a particularly important value for well-being as they can provide support to each other by just listening, advising, taking over tasks or cheering each other up. Working together and making decisions as a team, with supervisors or for example with colleagues from other specialties, makes residents feel good and more satisfied with their job.

#### Learning at the workplace

It is a pleasant feeling when there is an accessible atmosphere in residency training. This makes me more inclined to ask questions, discuss things and encourages me to reflect on former situations. It is nice to notice that the supervisors also appreciate this.

The learning climate is extremely important for helping residents enjoy their job. When there is an open atmosphere wherein they are offered ample learning opportunities, the residents experience feelings of meaningfulness and satisfaction. In an accessible and safe atmosphere residents feel more invited to ask questions and discuss things. When residents rotate to another teaching site, which is part of their training program, they sometimes experience a temporary setback in regard to learning opportunities as they first have to again prove their competency in different clinical tasks. A resident noted this as undesirable and difficult. Courses and conferences are often seen as having both educational and entertainment value. The residents like to learn new things and to experience progress in their knowledge and skills, making them enjoy their work and feel more confident. However, preparing for presentations and studying for exams is often seen as impinging on precious spare time. Assessments in residency training are sometimes experienced as a mental burden since the residents worry about it in advance. However, these can also boost energy if the results are positive or if the resident receives valuable feedback.

#### Dealing with the organization

What really takes energy is the logistics in this hospital. And the computer system is driving me crazy as well. For cardiology you have to report in program A and for neurology in program B, that kind of thing. It is impossible to work with; it frustrates me. They should throw away this computer system and some other logistical matters.

Duty hours, especially the nightshifts, are sometimes experienced as a factor negatively influencing the residents’ mood. Residents note sleeping less and feeling more tired and less enthusiastic about their job during this period. Slow computer systems and inefficacious logistics where the resident, for example, needs to make multiple phone calls and fill out a form just to order one diagnostic test are noted as irritating to residents. Administration is seen as one of the mandatory and less enjoyable tasks that takes a lot of time but can result in a feeling of calmness when completed.

### In personal lives

#### Dealing with personal characteristics

I am a perfectionist, and the thing is that I expect myself to be able to do everything. I want to be thoroughly prepared before starting my outpatient clinic day. That takes a lot of time and energy, especially in the beginning. […….] I want to do everything perfectly, and when that doesn’t work out it makes me feel angry.

The residents experience personal strength and power coming from a proactive and positive mindset, feeling energized and rested. These personal factors enable residents to deal with the demands in their working life, and mostly residents are aware of this. Personal characteristics that make it harder to deal with job demands are also noted by residents, such as perfectionism, being a brooder or finding it difficult to set boundaries. One resident attended a mindfulness course as a result of experiencing distress and felt this had resulted in more rest and a better overview, which improved her well-being.

#### Balancing work–life

I had a busy period with long working days. I quickly noticed that it had an impact on me personally. I didn’t have the time to recover from the workday like I used to. That influenced me and my private life in a way. I would arrive home later than I wanted. And I think my partner has suffered from it as I was more cranky than usual.

Residents note that their job takes up a significant amount of time and that they are aware of the need to be flexible with their spare time. Most residents seem to be fine with this as long as they enjoy their job and their functioning in it and if their private life enables it. They also note that they can handle busy working periods because they know it is temporary, and they feel they can be of value. However, the residents’ private time is also viewed as important because they derive energy from, for example, socializing with friends and family, playing sports, taking a mindfulness course or going on vacation. These “time outs” are crucial to recharge; residents return feeling fit and energized to perform their job. They feel supported in their private lives when they are able to vent work-related issues and feel appreciated by their loved ones.

## Discussion

In experiencing (un)well-being in relation to residents’ professional lives, the effort–reward (im)balance and perceived (in)sufficient autonomy were found to play a dominant role. Residents’ experiences of well-being were also found to be influenced in different ways in both their professional (delivering patient care, participating in teamwork, learning at the workplace, dealing with the organization) and personal (dealing with personal characteristics, balancing work–life) lives.

### Stress and well-being dependent on effort–reward (im)balance

The overarching experience of the influence of the balance between efforts and rewards on residents’ well-being is in accordance with previous work engagement and burnout research (Maslach et al. 2001; Bakker and Demerouti 2007). Jennings et al. also reported this balance as important for residents’ well-being (Jennings and Slavin 2015). A finding contrasting with the study from Jennings et al. is that our participating residents did not mention material compensation as a reward. This may be explained by the differences in residents’ financial situations after medical school and during residency training between the Netherlands and the United States. Over 80% of American medical students are in debt when they graduate with an average of USD $86,870 (Rosenblatt and Andrilla 2005). This is in contrast with Dutch students having an average debt of USD $17,000 after graduation (Broersen 2012).

The comments from residents that a compliment from a supervisor or the energy they receive from performing their job well makes them able to handle the busy outpatient clinic day or a negative event are in line with the literature about the job demands and resources model showing that resources are capable of buffering the impact of job demands (Bakker et al. 2005; Bakker and Demerouti 2007). The job demands and resources model is often used to describe demands and resources influencing work-related well-being (Bakker and Demerouti 2007). This model also shows that personal resources play a significant role in perceptions of stress and experiencing well-being (Xanthopoulou et al. 2007, 2009). This is in accordance with our residents experiencing that intrinsic motivation and optimism positively influence their felt energy and thereby their experienced well-being. However, as described in our study, an experienced imbalance between efforts and rewards seems to negatively impact residents’ well-being. For example, residents who experience incomprehension from peers and inadequate supervisory support report experiencing higher levels of stress.

### Perceived autonomy impacting well-being experiences

Previous studies have reported autonomy as important for intrinsic motivation and work-related well-being (Bakker and Demerouti 2007; Maslach et al. 2001; Ryan and Deci 2000). In the job demands and resources model, autonomy is also described as one of the job resources that can positively influence work-engagement and work-related well-being. This study also confirms these findings to be valid for the experiences of the residents. As an addition to this model, we found that the value of autonomy is experienced in different situations. The residents reported that experiencing sufficient autonomy in their various professional roles as caregivers (making treatment decisions) and learners (determine personal learning goals) and also in their private lives (scheduling spare time) is important to feeling good and performing well. Ryan and Deci found that conditions supportive of autonomy and competence can facilitate the intrinsic motivation, self-regulation and well-being of human beings (Ryan and Deci 2000). Therefore, enhancing these conditions appears to be valuable for the individual resident as well as the health care system as a whole.

The issue of autonomy in modern residency training reform is addressed by the introduction of the entrustable professional activity (EPA) concept (Ten Cate 2014; Mulder et al. 2010; Ten Cate et al. 2015). This concept allows supervisory faculty to make competency-based decisions about tasks or responsibilities that can be entrusted to a resident. As residents noted the importance of being assigned appropriate responsibilities, the use of EPAs might improve one’s feeling of autonomy and therefore one’s experienced well-being. Secondly, the current personalization of residency training allows for more autonomy. The ability to choose one’s own residency training schedule and define personal learning goals and learning strategies appeared to positively influence residents’ well-being. This is relevant within the context of self-directed learning (Grow 1991; Newman and Peile 2002). Active learners who have the opportunity to self-direct their own learning process seem to experience greater well-being. Additionally, exploring what well-being means for the individual resident could be a relevant part of the individualized residency training program.

### Ways that influence well-being

When residents talked about work-related well-being, various influencing experiences seemed to play a role. We could sort these experiences the residents noted during the interviews into ways that influence well-being in both their professional and personal lives. They reported how these ways in combination are responsible for their well-being experience. Residents who report, for example, enjoying their professional activities, the learning environment and their team also report experiencing overall good physical, mental and emotional well-being. The experienced ways that influence residents’ well-being resonate with research in positive psychology (Tiggelaar 2016; Seligman 2004; Seligman and Csikszentmihalyi 2014). Tiggelaar, for example, described three pillars of work-related happiness: enjoyable, good and meaningful work (Tiggelaar 2016; Seligman 2004; Seligman and Csikszentmihalyi 2014). These pillars could be identified in the residents’ experiences and the ways that influence well-being they described; the residents noted that working with kind and helpful colleagues as well as having pleasant supervisors positively influenced their well-being (enjoyable work). Additionally, delivering optimal patient care and developing oneself (good work) as well as meaning something to someone and being appreciated while delivering patient care (meaningful work) seemed to give the residents energy and had a positive impact on their well-being. In line with burnout research, the residents’ well-being could be negatively impacted when residents have negative experiences in regard to one or more of the described ways or when a negative experience continues for too long (Prins et al. 2007; Maslach et al. 2001). When well-being is negatively impacted, emotional and mental well-being are affected first and are followed by physical well-being when the experienced imbalance between efforts and rewards continues for too long.

### Strengths and limitations

By interviewing residents from various hospitals and specialties, we were able to sample a wide range of residents’ experiences, resulting in a thorough understanding of residents’ experienced well-being in relation to their professional life with the perceived influencers. This in-depth information about residents’ well-being gives us suggestions for improvement. Our study has also some notable limitations. We chose to interview the residents only once, however a longitudinal approach would have allowed us to also identify changes in well-being in relation to specific factors. Additionally, as we used a semi-structured interview guide to explore residents’ experiences and gave examples of well-being domains in our semi-structured interview guide, we cannot exclude whether this might have pre-determined the participants’ answers. Although we interviewed residents from many different kinds of specialties, not all specialties are included in this study. Consequently, we may have missed specific perspectives and experiences.

### Implications

As Hurst et al. also mentioned, residents are vulnerable in their demanding learning and working environments (Hurst et al. 2013). A perceived good balance between efforts and rewards and sufficient autonomy were found to be essential in regard to the residents’ experience of well-being. To create a greater sense of autonomy, residents could be supported by interventions. For example, successful interventions in increasing control over the dynamic work environment in health care are described by Dunn et al. (2007). Team-based peer support programs seem to be another option in regard to managing challenges in order to experience autonomy and improve well-being (Le Blanc et al. 2007; Shapiro and Galowitz 2016).

Additionally, it seems helpful to offer or make residents aware of resources that increase resilience and stabilize or enhance well-being as these might tilt the effort–reward balance more positively toward rewards (Epstein and Krasner 2013; Kreitzer and Klatt 2016; Jennings and Slavin 2015; West et al. 2016). Interesting options include focusing on how to balance energy expenditure and energy recovery or doing mindfulness training as this seems to have a positive impact on one’s experienced well-being (Schwartz and McCarthy 2007; Krasner et al. 2009; Lases et al. 2016). Expectation management for (starting) residents is in this context also crucial (Yeo et al. 2009). When residents encounter a different work environment and different job content than expected, they can end up disappointed and, experience a decrease in well-being and quit residency training, as our data also revealed. Good, solid preparation and clear communication before starting as well as during residency training seem to be of great value. Further research on the best interventions or ways for residents to stay well, improve their effort–reward balance or enhance autonomy is needed.

## Conclusion

In the residents’ well-being experiences, the effort–reward balance and perceived autonomy are crucial. Additionally, ways that influence residents’ well-being are identified in both their professional and personal lives. These dominant experiences and ways that influence well-being could be key factors for developing and/or providing interventions and residency training adaptations for enhancing residents’ well-being.

## Appendix

See Table 2.

**Table 2:** Semi-structured interview guide

Key questions	Topics
1. How did you experience your work today (/last working day)?	Contents/tasks
	Experiences/feelings
	Example of a good/enjoyable day
We’ll try to delve deeper into your well-being. Globally we could divide well-being into different domains—e.g. physical, mental and emotional well-being—which I would like to address in the following section of the interview	
2. Keeping a period of 4 weeks in mind: How do you experience your	
	Well-being on a professional level
Physical well-being?	Well-being on a private level
Mental well-being?	Influencing factors/energy takers/energy givers
Emotional well-being?	Relation of well-being and professional life as a resident
